# Intelligence

**Published:** 1842-04-01

**Authors:** 


### CORRESPONDENCE, INTELLIGENCE, &c.

### UNIVERSITY OF NEW YORK.

We are glad to see that the medical department of this rising University has assumed an aspect of great strength and importance. It embraces the highly respectable names of Theodore Frelinghuysen, Valentine Mott, Granville Sharp Pattison, J. Revere, Martyn Paine, G. Bedford, J. W. Draper, &c. &c. We wish it every success, and feel proud at seeing our talented countryman, Dr. G. S. Pattison, enrolled on the distinguished list of Professors.

---

We regret that we are unable to comply with the learned Editors of the "Medicinischen Correspondentlett," as we have no means of transmitting our Journal to Leipzig or Erlangen. If they can point out any person in London to whom we can transmit the Journal, we will do so with pleasure.

---

We regret to inform many valued correspondents that, in future, we are unable to insert ORIGINAL COMMUNICATIONS, as our Review and Periscopic departments require the whole extent of our pages.

